# Schizophrenia, Nutrition and Choices in Kilojoules (SNaCK): protocol for a feasibility and acceptability randomised controlled trial of two dietary interventions

**DOI:** 10.1192/bjo.2025.10070

**Published:** 2025-07-22

**Authors:** Donni Johnston, Urska Arnautovska, Andrea Baker, Ingrid J. Hickman, Hannah L. Mayr, Nicole Korman, Wolfgang Marx, Eryn Murray, Nicola Warren, Sarah Weighell, Veronica De Monte, Gemma McKeon, Dan Siskind, Scott B. Teasdale

**Affiliations:** Faculty of Health, Medicine and Behavioural Sciences, University of Queensland, Brisbane, Queensland, Australia; Metro South Addiction and Mental Health Services, Logan Central, Queensland, Australia; Metro South Addiction and Mental Health Services, Woolloongabba, Queensland, Australia; Queensland Centre for Mental Health Research, Wacol, Queensland, Australia; Department of Nutrition and Dietetics, Princess Alexandra Hospital, Woolloongabba, Brisbane, Queensland, Australia; Centre for Functioning and Health Research, Metro South Health, Brisbane, Queensland, Australia; Metro South Addiction and Mental Health Services, Coorparoo, Queensland, Australia; Institute for Mental and Physical Health and Clinical Translation (IMPACT), Food & Mood Centre, School of Medicine, Deakin University, Barwon Health, Geelong, Victoria, Australia; West Moreton Health Psychology, Wacol, Queensland, Australia; Child Health Research Centre, University of Queensland, South Brisbane, Queensland, Australia; Discipline of Psychiatry and Mental Health, School of Clinical Medicine, University of New South Wales, Sydney, New South Wales, Australia

**Keywords:** Mental disorders, schizophrenia, diet, cooking, nutrition

## Abstract

**Background:**

Individuals with schizophrenia experience significantly higher rates of chronic physical health conditions, driving a 20-year reduction in life expectancy. Poor diet quality is a key modifiable risk factor; however, owing to side-effects of antipsychotic medication, cognitive challenges and food insecurity, standard dietary counselling may not be sufficient for this population group.

**Aim:**

To evaluate the feasibility, acceptability and preliminary effectiveness of two dietary interventions – pre-prepared meals and meal kits – for individuals with schizophrenia.

**Method:**

The Schizophrenia, Nutrition and Choices in Kilojoules (SNaCK) study is a 12-week, three-arm, cross-over, randomised controlled trial. Eighteen participants aged 18–64 years diagnosed with schizophrenia or schizoaffective disorder will be recruited from community mental health services in Australia. Participants will be randomised to receive pre-prepared meals, meal kits or a supermarket voucher as a control, crossing-over at the end of weeks 4 and 8, so that all participants experience all three study arms. Primary outcomes include feasibility (recruitment rate and retention, number of days participants use pre-prepared meals or meal kits, adherence to meals as prescribed, difficulty in meal preparation and meal wastage) and acceptability (meal provision preference ranking and implementation) of the nutrition interventions. Secondary outcomes include the effects of the intervention on metabolic syndrome components, dietary intake, quality of life and food security measures.

**Conclusions:**

Feasible, acceptable and effective dietary interventions for people with schizophrenia are urgently needed. Findings from this trial will inform future larger randomised controlled trials that have the potential to influence policy and improve health outcomes for this vulnerable population.

People living with schizophrenia experience chronic physical health conditions at rates 2–5 times higher than the general population,^1^ with cardiovascular disease making the greatest contribution to the 10- to 16-year gap in life expectancy.^2^ Drivers of these disparities can be wide-ranging. A key modifiable risk factor is poor diet, frequently characterised in this population group by excessive dietary intake of poor nutritional quality.^3^ Side-effects of antipsychotic medication (and other psychotropics) often include increased hunger,^4^ food cravings and disordered eating behaviours^5^ such as binge eating, while also potentially having sedative effects.^6^ Cognitive impacts of the illness, such as impairments in executive functioning, facilitate eating disinhibition^7^ and may affect a person’s knowledge, confidence, skills and motivation to plan and prepare nutritious meals.^8^ Further, food access is frequently an issue, given the limited income of many people living with schizophrenia.^9^

Lifestyle interventions are considered an effective means of improving metabolic health in this population group; however, without adequate resources, they are often affected by uptake, adherence and attrition issues, particularly in people who experience more severe clinical symptoms.^10^ Pre-prepared meals and meal kits require less effort and planning compared with the complex cognitive process of planning, shopping and preparing a meal and may enable and empower people with schizophrenia to independently prepare and consume healthier meals. A recent pilot study (*n* = 24), including people with depression and food insecurity who were provided with a weekly box of fresh fruit and vegetables, found that the intervention was acceptable to participants, with two-thirds of participants showing improvements in depression at project end.^11^ To our knowledge, the feasibility and impact of providing healthy pre-prepared meals or meal kits has not previously been investigated in a population of individuals living with mental illness.

Here, we aimed to test the feasibility, acceptability and preliminary efficacy of dietary interventions using pre-prepared meals and meal kits among people living with schizophrenia.

## Method

### Study design

**Fig. 1:**
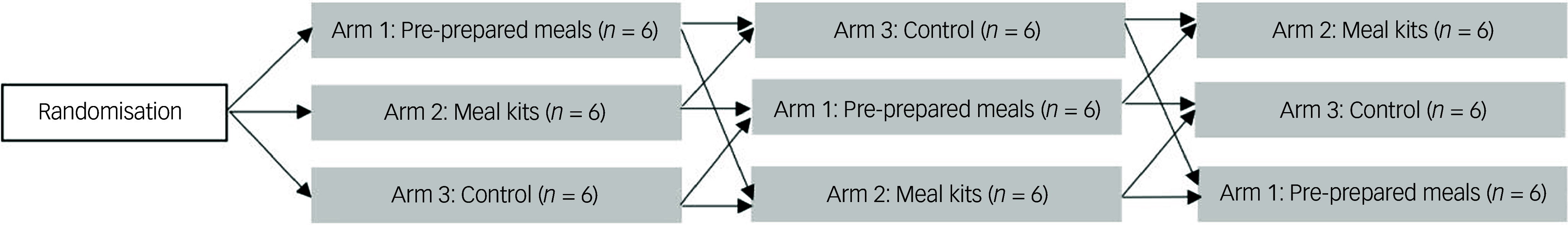
Participant randomisation for each 4-week arm.

The SNaCK study is a 12-week, three-arm, cross-over, three-arm randomised controlled trial (RCT) (4 weeks per study arm) using a mixed-methods evaluation to test the feasibility, acceptability and preliminary efficacy of two dietary interventions compared with an active control condition among people with schizophrenia or schizoaffective disorder (Fig. 1). This trial was registered with the Australian and New Zealand Clinical Trials Registry (trial ID: ACTRN12623001024639) and reported according to the SPIRIT-Outcomes 2022 checklist.^12,13^

### Patient and public involvement

In preparation of this protocol, two consumers from a community-based rehabilitation service living with schizophrenia were engaged to inform the proposed study design, including assessment measures. The consumers trialled both the meal kits and the pre-prepared meals and provided feedback on their experience, which was incorporated in the finalised protocol and study design.

### Setting and study population

Eighteen eligible participants, aged 18–64 years, will be recruited through public community mental health rehabilitation settings (Community Care Units) at Metro South Hospital and Health Services, Australia. Participants will complete all three study arms, per recommendations for participatory design and user testing activities.^14^

Consumers will be invited to participate in the study if they have a diagnosis of schizophrenia or schizoaffective disorder (DSM-5) and body mass index (BMI) ≥25 kg/m^2 15^ at baseline, have had <5% body weight increase or loss in the previous 3 months and live in independent accommodation with access to a kitchen with relevant utensils and equipment. All participants must have capacity to consent, be able to follow the study instructions and procedures, and provide their informed consent.

Capacity will be determined by collaboration between the treating team and delegated research staff, in compliance with the National Health and Medical Research Council (NHMRC) National Statement on Ethical Conduct in Human Research.^16^ All research staff are experienced in assessing the capacity of people with severe mental illness to provide informed consent. Ability to provide informed consent will be overseen by the participant’s treating psychiatrist.

Consumers will be excluded from the study if they are experiencing acute psychiatric symptoms requiring immediate hospital admission, have obesity induced by another endocrinologic disorder (e.g. Cushing syndrome, untreated hypothyroidism), have had previous surgical treatment of obesity, have a current or prior history with risk of relapse of a severe eating disorder, have dietary allergies that preclude safe consumption of provided meals or foods, or are living with someone who prepares meals for them.

### Intervention procedures

The two dietary interventions will be: (a) pre-prepared meals (i.e. Lite n’ Easy) and (b) meal kits (i.e. EveryPlate box with meal ingredients and preparation and/or cooking instructions). These two interventions were chosen on the basis of a review of options for pre-prepared meals and meal kits available at the time in Australia, conducted by a dietitian, which was further discussed with the research team.

All participants will receive 15 min in-person general nutrition and food safety education by a nutritionist at baseline, including hard copy resources on how to safely store and prepare food, how to identify and follow ‘best before’ and ‘use by’ dates, and information on healthy meal options aligned with the Australian Dietary Guidelines (2013).^17^

The order of the three arms that each participant will receive will be determined using a computer-generated randomisation table by an independent biostatistician. The data analyst will be blinded to randomisation to avoid bias, but the rest of the research team will be unblinded. This will allow the research team to ensure the timing of the ordering (dietary interventions) is aligned with the participant randomisation arm.

#### Pre-prepared meals

Lite n’ Easy is a commercial company in Australia that produces and delivers pre-prepared meals. Lite n’ Easy was chosen owing to the diversity of frozen meal options, delivery capability, dietitian involvement in menu design, and access to comprehensive meal nutrition information, and because it is an existing National Disability Insurance Scheme (NDIS)-approved provider.

In Australia, the NDIS provides funding and support for people with a disability, including those with schizophrenia. In certain cases, where the illness (or disability) means planning meals and following multi-step instructions is troublesome, the NDIS may subsidise the delivery of pre-prepared meals for a period.

#### Meal kits

EveryPlate is a commercial company in Australia. EveryPlate was selected as it provided the simplest recipes (four-step recipes) using affordable, known ingredients with the least requirements for existing pantry items or complex equipment compared with other providers. Although not currently eligible as an NDIS-subsidised meal option, meal kits were chosen as they remove the complex steps of meal planning and grocery shopping while also supporting the development of basic cooking skills with step-by-step guides, making cooking with fresh ingredients a more viable option.

For each dietary intervention phase, participants will receive one meal daily, delivered weekly, for 4 weeks. A similar amount of kilojoules (kJ) will be provided per week by the meals, with Lite n’ Easy and EveryPlate meals approximately matched for kJ content (between 2600 and 2900 kJ per meal). Meal ordering will be conducted by a nutritionist and based on nutritional quality, ease of preparation (meal kits) and participant food preference. Participant food preferences, such as a preference for major protein sources (meat, poultry, seafood or vegetarian), cuisines and spices, will be collected at baseline, and participants will be encouraged to inform the research team if their food preferences change or are not appropriately reflected in the meal selection.

#### Control

Participants in the active control phase will receive a weekly $100 AUD supermarket voucher for 4 weeks. This was chosen to ensure study participant retention during the control arm and relatively equal monetary value in each arm so as not to influence the food security measure.

### Trial visits, assessments and outcome measures

Study visits and assessments will follow the schedule outlined in Table 1. The clinical measures will be undertaken by experienced mental health clinicians trained in administering the study measures and supervised by the research manager. Clinical measures will be administered at the participant’s choice of either the mental health clinic or their home residence.

**Table 1:** Schedule of visits and assessments

Assessments														
Visit	0	1	2	3	4	5	6	7	8	9	10	11	12	13
	Screening	Baseline												
Week		0	1	2	3	4	5	6	7	8	9	10	11	12
Study period (12 weeks)														
Screening and consent														
Assessment of current medication	x	x	x	x	x	x	x	x	x	x	x	x	x	x
Informed consent	x													
Ongoing capacity	x	x	x	x	x	x	x	x	x	x	x	x	x	x
Inclusion and/or exclusion criteria	x													
Safety														
Adverse events		x	x	x	x	x	x	x	x	x	x	x	x	x
Clinical profiling														
Demographics (e.g. age, gender, indigenous status, marital status, education, employment)		x												
Medical history and medications (e.g. mental health conditions, physical health conditions, duration of illness)		x												
PANSS total		x												
Physical activity (SIMPAQ)		x												
Trail Making Test		x												
CVLT-III short form		x												
Rey Complex Figure Test		x												
Tower of London		x												
GAF		x												
Feasibility														
Food adherence^a^														
Meal adherence			x	x	x	x	x	x	x	x	x	x	x	x
Meal wastage			x	x	x	x	x	x	x	x	x	x	x	x
Meal preparation difficulty			x	x	x	x	x	x	x	x	x	x	x	x
Acceptability survey^b^					x				x					
Implementation of interventions (via semi-structured qualitative interview)						x				x				x
Efficacy														
Height		x												
Body weight (kg), BMI		x				x				x				x
Waist circumference and hip/waist ratio		x				x				x				x
Heart rate		x				x				x				x
Blood pressure		x				x				x				x
Dietary intake (24 h diet recall)		x			x				x				x	
Quality of life (SF-36)		x				x				x				x
Food insecurity (HFIAS)		x				x				x				x
Food knowledge, acquisition, and preparedness		x				x				x				x

BMI, body mass index; SF-36, Short Form Survey; HFIAS, Household Food Insecurity Access Scale; PANSS, Positive and Negative Syndrome Scale; SIMPAQ, Simple physical Activity Questionnaire; CVLT-III, California Verbal Learning Test 3rd Edition; GAF, Global Assessment of Functioning.

a Delivery of the two interventions and adherence to the instructions of both intervention options will not be completed for the control arm.

b The acceptability survey will not be completed for the control arm.

The measures relevant to the primary objective of the trial (i.e. assessment of feasibility and acceptability of two dietary interventions) will be collected through a checklist which participants will be requested to complete daily during the 4 weeks of each intervention arm and weekly for the control arm. To assist participants with completing the dietary checklists, research staff will send SMS reminders to participants and collect the checklists weekly.

This information will be complemented with data obtained from: (a) a semi-structured interview, conducted after participation in both dietary interventions and the control condition (week 12), asking participants about their views and experiences of each intervention; and (b) an acceptability survey, completed in week 3 to align with the 24-h diet recall visit.

### Outcome variables

#### Clinical profiling

The following measures will be conducted at baseline to establish a profile of the participant cohort and account for potentially confounding factors.

- Demographics and medical history: participant age, gender, indigenous status, marital status, education, employment, smoking status, current mental and physical health diagnosis, duration of illness, length of time spent in a mental health rehabilitation setting and current medications.
- Positive and Negative Symptom Scale: subscales including positive, negative and general psychopathology, a widely used scale for measuring symptom severity of people with schizophrenia.^18^
- Simple Physical Activity Questionnaire: a five-item questionnaire developed as a clinical tool to assess physical activity and sedentary behaviour in people living with mental illness, using an interview format to provide a snapshot of a 24-h period estimate of time in bed, minutes of structured exercise participation, and incidental or non-structured physical activity.^19^
- Trail Making Test: a neuropsychological test of attention, sequencing, cognitive flexibility, visual scanning, processing speed and motor function. The test can provide information about a person’s ability to alternate attention or shift mental set.^20^
- California Verbal Learning Test brief form: a brief screening measure of episodic verbal learning and memory, which demonstrates sensitivity to a range of clinical conditions.^21^
- Rey Complex Figure Test and Recognition Trial: captures information about five domains of neuropsychological functioning. Visuospatial recall memory and recognition memory are the primary reasons to give this measure.^22^
- Tower of London: a neuropsychological measure of planning ability that is widely used in clinical and research contexts.^23^
- Global Assessment of Function: a numeric scale (1 to 100) used by mental health clinicians and physicians to rate subjectively the social, occupational and psychological functioning of adults.^24^

#### Feasibility

Feasibility will be determined by self-reported: (a) meal adherence, (b) meal wastage and (c) meal preparation difficulty using a dietitian-designed daily checklist (Supplementary File 1 available at https://doi.org/10.1192/bjo.2025.10070) to record consumption of pre-prepared meals or meal kits as intended.

#### Acceptability

Acceptability will be determined by recruitment rate and retention, completion of the acceptability survey and semi-structured qualitative interviews. The acceptability survey will determine the participants’ perceived acceptability of each meal intervention using a nine-item, five-point Likert scale survey (Supplementary File 2), with each item aligned with a validated construct of acceptability from the Theoretical Framework of Acceptability.^25^ In addition, the participant will be given an opportunity to provide a short open-ended clarification for each score. The semi-structured interviews will explore participant experience of and preference for each intervention, questions informed by the Acceptability of Intervention Measure, Intervention Appropriateness Measure, and Feasibility of Intervention Measure.^26^ In addition, participants will be asked to rank all three arms of the study in order of preference.

#### Efficacy

Secondary outcome measures (i.e. exploration of the preliminary efficacy of two dietary interventions) will be assessed at the start (baseline) and at the end of each 4-week block, except for dietary intake, which will be assessed at baseline and week 3 of each arm (a time frame chosen to reduce participant burden in week 4). Exploratory efficacy will be evaluated using the following measures.

Physical health measures: weight will be recorded to the nearest 0.1 kg with calibrated Ade M322600 portable scales, with the same pair of scales used throughout the trial. Height, without shoes, will be measured using a tape measure mounted on the wall, with an accuracy of 0.1 cm. BMI will be calculated as weight (kg)/height (m^2^). Waist circumference will be measured halfway between the lower border of the ribs and the iliac crest in a horizontal plane. Hip circumference will be measured around the widest portion of the buttocks in a horizontal plane. Both measures will be to the nearest 0.5 cm using a non-stretchable measuring tape. Participants will be asked to remove belts or garments that may change the shape of their bodies. Where possible, the same research staff will take measurements for a participant at baseline and follow-up time points for consistency. Waist-to-hip ratio will be calculated as waist circumference (cm) divided by hip circumference (cm). Systolic and diastolic blood pressure will be measured manually at all visits using an EliteCare Traditional Sphygmomanometer (accuracy: 0.5 mm Hg) on the left arm while sitting. Participants will be asked to avoid caffeine, smoking and physical activity for 30 min before the measurements. Pulse will be recorded after 5 min of seated rest.

24-h multi-pass dietary recall: a five-phase face-to-face assessment of dietary intake conducted by a nutritionist or dietitian. The participant is guided to iteratively provide detail about each food and drink consumed within the previous 24-h period (modelled on the US Department of Agriculture’s Automated Multi-Pass Method.^27^). The 24-h recall will be entered into FoodWorks Online Professional (version 2, Xyris, Brisbane, Australia) for nutritional analysis including energy, macronutrient, sodium, and core and discretionary food group intake.

Short Form 36 Health Survey: a self-rated survey, validated for people with schizophrenia,^28^ measuring overall quality of life and covering general health, activity level, and emotional and somatic complaints with associated disability. The measure produces two component scores: the physical component summary and mental component summary. A high score represents a more favourable health state; the lowest and highest possible scores are 0 and 100, respectively.^29^

Household Food Insecurity Access Scale (HFIAS): a nine-question food security measurement tool, assessing feelings or behaviour around food anxiety, food quality and food quantity over the previous 30 days.^30^ The HFIAS has been used in several countries and appears to distinguish food-insecure from food-secure households across different cultural contexts. The information generated by the HFIAS can be used to assess the prevalence of household food insecurity (access component) and to detect changes in the food insecurity situation of a population over time.^31^

Food knowledge, acquisition and preparedness: a self-administered quantitative questionnaire will be used to assess cooking confidence (self-efficacy) and cooking and eating behaviours. Cooking confidence (self-efficacy) is evaluated using five questions that assess participants’ confidence in cooking skills necessary for basic home cooking. These skills are rated on a scale from 1 to 5, with 1 representing ‘not at all confident’ and 5 indicating ‘extremely confident’. An overall confidence score is calculated by summing the individual item scores.

Cooking and eating behaviours will be evaluated using six questions. Frequency of cooking the main meal from basic ingredients, frequency of eating ready-made meals, and frequency of eating vegetables with the main meal are measured on a scale from 0 (never) to 7 (daily); daily vegetable intake is measured on a scale from 0 (no servings per day) to 6 (six or more servings per day); and number of servings of fruit per day and frequency of eating takeaway meals are measured from 0 (never) to 5 (five or more times per week).^32^

### Data management

Data will be collected and stored according to *Good Clinical Practice*. A screening log will be used to track potential participants, consent, eligibility, withdrawals and completion as per the Consolidated Standards of Reporting Trials requirements.^33^ De-identified, hard copy case report forms (CRF) with questionnaires and clinical data will be stored securely at the research team site. CRF data will be entered into REDCap, a secure web-based application, and exported into statistical analyses software (IBM SPSS version 29).

Owing to this being a pilot trial, no data management committee is required. All adverse events and serious adverse event data will be reported to all study investigators, who will jointly decide whether a stopping procedure should be instigated.

### Data analyses

Basic tabular descriptive statistics will include sample size for each group, range of scores, means, standard deviations and coefficient of variations for continuous variables with reasonably symmetrical distributions, or median and interquartile range for highly skewed variables. Differences in baseline mean symptom severity (measured with the Positive and Negative Symptom Scale and Global Assessment of Function) will be assessed across the three groups using unstandardised mean differences.

Primary outcome measures will be assessed against predetermined criteria for feasibility and acceptability as listed in Table 2. Primary and secondary outcomes will be assessed using *t*-tests and Mann–Whitney *U*-test for continuous variables. Categorical variables will be assessed using chi-squared tests by comparing the baseline scores for all participants randomised to one arm with end-point scores (at week 4) of all participants for that arm and in the same way for the other two study arms. This within-intervention analysis will then be extended to compare the same outcomes across the three arms using analysis of variance. Potential confounders such as gender, age, physical comorbidities and BMI will be considered.

**Table 2:** Primary outcome measures and criteria for success

Feasibility	Intervention	Data	Collection method	Success criteria
Meal adherence	Pre-prepared meals	Proportion of pre-prepared meal eaten.	Checklist	Half or more of the meal was eaten ≥80% of the days.
	Meal kit	Did you follow the recipe? Did you split the recipe into two servings? Did you eat the prescribed portion of the meal kit meal today?	Checklist	Recipe followed at least 50% of time (six of 12 recipes). Of recipes attempted, food split into two meal servings 100% of time. Half or more of the meal serving was eaten ≥80% of the days.
	Control	Proportion of voucher spent on food?	Checklist	3 of 4 weeks ‘all’ voucher spent on food.
Meal wastage	Pre-prepared meals	If prepared meals were uneaten,– what happened to leftovers?	Checklist	Of meals prepared, some or all food ‘went in bin/remained uneaten’ <25% of the time.
	Meal kit	If recipe not followed what happened to unused ingredients?	Checklist	For each kit, unused ingredients went in the bin or remained uneaten ≤1 time.
		If unused ingredients at end of week, what happened to them?	Checklist	For each kit, unused ingredients went in the bin or remained uneaten ≤1 time.
		If you didn’t eat all of your meal portion, what happened to the leftovers?	Checklist	Of recipes prepared, some or all food went in the bin or remained uneaten <25% of the time.
Food preparation difficulty	Pre-prepared meals	How difficult was the meal preparation?	Checklist	Considered easy or moderate ≥80% of the time.
	Meal kit	How difficult was the meal preparation?	Checklist	Considered easy or moderate ≥80% of the time.
	Control	How difficult was meal preparation this week?	Checklist	Considered hard more frequently than in either meal provision group
Acceptability	Pre-prepared meals and meal kit	Participants preference for using each meal option	Semi structured interview	Ranking of two options for meal provision. Open-ended question
	Pre-prepared meals and meal kit	Participants rating of acceptability across nine items.	Acceptability survey	≥4 in ≥70% of participants (feasible), 55–69% participants (risks identified), <55% participants (not feasible)

Correlations between cognition scores taken during baseline clinical profiling and indicators of capacity to prepare meals, such as self-reported meal preparation difficulty, will be explored using Cronbach’s alpha.

Qualitative interviews will be recorded and transcribed by the research staff. The qualitative interview data will be analysed using a reflexive thematic analysis^34^ approach in NVivo 14. D.J. and U.A. will familiarise themselves with the data by reading and discussing responses. D.J. and U.A. will take both inductive and deductive approaches by exploring responses while organising them around three quantitative questions related to acceptability and feasibility. Twenty per cent of responses will be jointly coded to develop a thematic framework, after which D.J. will code the remainder of responses. D.J. and U.A. will iteratively review and define themes using triangulation of data with other measures. All authors will be involved in producing the final report and will review and discuss the themes.

### Participant safety

Participation in this trial is low risk as consuming pre-prepared meals or preparing meals with fresh ingredients is deemed safe. Participants are screened for any known allergies before consenting, and reasonable food preferences will be catered. There are risks that are associated with participation in any trial (confidentiality breach, discomfort answering research questions, no benefit from the intervention product; see the supplementary material for detailed risks). A risk identification, evaluation and management plan (Supplementary File 3) will be used to ensure that risks and uncertainty are appropriately managed for the duration of the study.

### Ethics and dissemination

The authors assert that all procedures contributing to this work comply with the ethical standards of the relevant national and institutional committees on human experimentation and with the Helsinki Declaration of 1975, as revised in 2008.^35^ All procedures involving human participants and/or patients were approved by Metro South Human Research Ethics Committee (HREC/2023/QMS/99814). All study participants will be assigned a unique identifier, and no identifying information is to be entered by the research team on any CRF, document or biological specimen. The research team will not disclose the identity of any research participant to a third party, unless permitted or required by law. The research team will retain study documentation for at least 15 years or the maximum time frame as determined by local regulations, whichever is the longest. Audio recordings will be destroyed according to the local Health and Hospital Service destruction policies upon completion of the project.

Participants that have experienced adverse events (e.g. injuries related to cooking and/or food preparation that require first aid or health professional attention) during the trial will be monitored until resolution and/or we will liaise with the treating team to optimise ongoing care as appropriate. If any participant experiences a post-study adverse event that is deemed related to the intervention, this will be managed until completely resolved. Study findings will be disseminated through peer-reviewed publications and conference presentations.

## Discussion

### Dietary interventions for people living with schizophrenia

Dietary interventions, often provided as part of broader lifestyle interventions, have proven effective for metabolic outcomes post-intervention for people living with schizophrenia.^36^ However, dietary counselling and education interventions may not be appropriate and sustainable for all people living with schizophrenia as a sole intervention.

The provision of pre-prepared meals and/or meal kits to the person’s home may bypass frequent barriers to healthy eating, such as reduced motivation^37^ and non-attendance at appointments,^38^ a lack of access to adequate nutritious foods stemming from financial constraints,^9^ social exclusion and stigma,^39^ difficulties in grocery shopping^40^ and/or barriers to using foods at home owing to inadequate food storage and cooking facilities, and cognitive barriers related to planning and preparing nutritious meals.^10^ Long-term reliance on pre-prepared meals or meal kits may diminish a person’s independence over time by reducing opportunities to develop or maintain skills such as meal planning, grocery shopping and, in the case of pre-prepared meals, cooking. Independence and sense of agency should be included as outcomes in larger trials. Further, a participatory approach to developing implementation strategies for nutrition programmes that potentially involve food provision will help to manage these risks strengthening the translation of study findings into diverse real-life settings.

### Potential for real-world impact

Our planned trial is timely, and, although its findings may inform practice, its primary purpose is to inform the design of RCTs that aim to assess interventions for metabolic health in people living with severe mental illness such as schizophrenia. Specifically, this study is intended to optimise trial protocols for both dietary and drug interventions by identifying how best to implement dietary interventions and/or maintain diet stability or security during drug interventions whereby variation in dietary intake may affect outcomes.

In Australia, there is potential for this study to affect insurance policy for individuals whose illness or disability makes meal planning and following multi-step instructions challenging. The NDIS covers 70% of meal costs for those with long-term disabilities. However, a lack of research in this area means current access and options are limited. This study contributes to the currently very limited scope of scientific evidence regarding the provision of meals and/or meal kits as an intervention for people with schizophrenia. It has the potential to build evidence on more appropriate and feasible meal provision for a population group that experiences some of the most adverse diet-related health outcomes globally.

### Limitations

A key limitation of this study is that as a pilot, it is not adequately powered to detect clinical efficacy. In addition, the nature of the intervention prevents blinding of participants and research staff to the diet intervention or control arm. To reduce any potential impact on study outcomes, participants and their treatment teams are informed of their next intervention 1 week before it begins. Although this lack of blinding may have some effect, it is unlikely to significantly influence our primary outcomes. All participants will experience both interventions and the control condition, providing an appropriate scenario to assess the feasibility and acceptability of the intervention, as well as the design needs for a well-powered efficacy study and subsequent implementation studies.

## Supporting information

Johnston et al. supplementary material 1 Johnston et al. supplementary material

Johnston et al. supplementary material 2 Johnston et al. supplementary material

Johnston et al. supplementary material 3 Johnston et al. supplementary material

## Data Availability

The data that support the findings of this study are available from the corresponding author upon reasonable request.

## Supplementary material

The supplementary material is available online at https://doi.org/10.1192/bjo.2025.10070

## References

[1] Correll CU , Solmi M , Veronese N , Bortolato B , Rosson S , Santonastaso P , et al. Prevalence, incidence and mortality from cardiovascular disease in patients with pooled and specific severe mental illness: a large-scale meta-analysis of 3,211,768 patients and 113,383,368 controls. World Psychiatry 2017; 16: 163–80. 28498599 10.1002/wps.20420 PMC5428179

[2] Lawrence D , Hancock KJ , Kisely S. The gap in life expectancy from preventable physical illness in psychiatric patients in Western Australia: retrospective analysis of population based registers. BMJ 2013; 346: f2539. 23694688 10.1136/bmj.f2539 PMC3660620

[3] Teasdale SB , Ward PB , Samaras K , Firth J , Stubbs B , Tripodi E , et al. Dietary intake of people with severe mental illness: systematic review and meta-analysis. Br J Psychiatry 2019; 214: 251–9. 30784395 10.1192/bjp.2019.20

[4] Blouin M , Tremblay A , Jalbert ME , Venables H , Bouchard RH , Roy MA , et al. Adiposity and eating behaviors in patients under second generation antipsychotics. Obesity 2008; 16: 1780–7. 18535555 10.1038/oby.2008.277

[5] Sankaranarayanan A , Johnson K , Mammen SJ , Wilding HE , Vasani D , Murali V , et al. Disordered eating among people with schizophrenia spectrum disorders: a systematic review. Nutrients 2021; 13: 3820. 34836076 10.3390/nu13113820 PMC8618287

[6] Stroup TS , Gray N. Management of common adverse effects of antipsychotic medications. World Psychiatry 2018; 17: 341–56. 30192094 10.1002/wps.20567 PMC6127750

[7] Elman I , Borsook D , Lukas SE. Food intake and reward mechanisms in patients with schizophrenia: implications for metabolic disturbances and treatment with second-generation antipsychotic agents. Neuropsychopharmacology 2006; 31: 2091–120. 16541087 10.1038/sj.npp.1301051

[8] Aubin G , Stip E , Gélinas I , Rainville C , Chapparo C. Daily functioning and information-processing skills among persons with schizophrenia. Psychiatr Serv 2009; 60: 817–22. 19487353 10.1176/ps.2009.60.6.817

[9] Teasdale SB , Müller-Stierlin AS , Ruusunen A , Eaton M , Marx W , Firth J. Prevalence of food insecurity in people with major depression, bipolar disorder, and schizophrenia and related psychoses: a systematic review and meta-analysis. Crit Rev Food Sci Nutr 2023; 63: 4485–502. 34783286 10.1080/10408398.2021.2002806

[10] Teasdale SB , Samaras K , Wade T , Jarman R , Ward PB. A review of the nutritional challenges experienced by people living with severe mental illness: a role for dietitians in addressing physical health gaps. J Human Nutr Dietet 2017; 30: 545–53. 10.1111/jhn.12473 28419586

[11] Engebretsen B , Kane A , Laroche H. The Food Box Pilot. Ann Family Med 2022; 20: 179. 10.1370/afm.2769 PMC8959739 35346932

[12] Chan AWTJ , Altman DG , Laupacis A , Gøtzsche PC , Krleža-Jerić K , Hróbjartsson A , et al. SPIRIT 2013 statement: defining standard protocol items for clinical trials. Ann Intern Med 2013; 158: 200–7. 23295957 10.7326/0003-4819-158-3-201302050-00583 PMC5114123

[13] Butcher NJ , Monsour A , Mew EJ , Chan A-W , Moher D , Mayo-Wilson E , et al. Guidelines for reporting outcomes in trial protocols: the SPIRIT-outcomes 2022 extension. JAMA 2022; 328: 2345–56. 36512367 10.1001/jama.2022.21243

[14] Bastien JMC. Usability testing: a review of some methodological and technical aspects of the method. Int J Med Inform 2010; 79: e18–23. 19345139 10.1016/j.ijmedinf.2008.12.004

[15] World Health Organization (WHO). *Physical Status: The Use and Interpretation of Anthropometry. Report of a WHO Expert Committee*. WHO, 1995. 8594834

[16] National Health and Medical Research Council. Australian Code for the Responsible Conduct of Research. Australian Research Council and Universities Australia, 2018.

[17] National Health and Medical Research Council. Australian Dietary Guidelines. National Health and Medical Research Council, 2013.

[18] Kay SR , Fiszbein A , Opler LA. The positive and negative syndrome scale (PANSS) for schizophrenia. Schizophr Bull 1987; 13: 261–76. 3616518 10.1093/schbul/13.2.261

[19] Rosenbaum S , Morell R , Abdel-Baki A , Ahmadpanah M , Anilkumar TV , Baie L , et al. Assessing physical activity in people with mental illness: 23-country reliability and validity of the simple physical activity questionnaire (SIMPAQ). BMC Psychiatry 2020; 20: 108. 32143714 10.1186/s12888-020-2473-0 PMC7060599

[20] Reitan RM. Trail Making Test: Manual for Administration and Scoring. Reitan Neuropsychology Laboratory, 1992.

[21] Delis D , Kramer J , Kaplan E , Ober B. California Verbal Learning Test, Adult Version (CVLT-II). The Psychological Corporation, 2000.

[22] Rey A. L’examen psychologique dans les cas d’encéphalopathie traumatique. (Les problems) [The psychological examination in cases of traumatic encepholopathy. Problems]. Arch Psychol 1941; 28: 215–85.

[23] Krikorian R , Bartok J , Gay N. Tower of London procedure: a standard method and developmental data. J Clin Exp Neuropsychol 1994; 16: 840–50. 7890819 10.1080/01688639408402697

[24] American Psychiatric Association (APA). Diagnostic and Statistical Manual of Mental Disorders (4th edn). APA, 1994.

[25] Sekhon M , Cartwright M , Francis JJ. Acceptability of healthcare interventions: an overview of reviews and development of a theoretical framework. BMC Health Serv Res 2017; 17: 88. 28126032 10.1186/s12913-017-2031-8 PMC5267473

[26] Weiner BJ , Lewis CC , Stanick C , Powell BJ , Dorsey CN , Clary AS , et al. Psychometric assessment of three newly developed implementation outcome measures. Implement Sci 2017; 12: 108. 28851459 10.1186/s13012-017-0635-3 PMC5576104

[27] Moshfegh AJ , Rhodes DG , Baer DJ , Murayi T , Clemens JC , Rumpler WV , et al. The US Department of Agriculture Automated Multiple-Pass Method reduces bias in the collection of energy intakes. Am J Clin Nutr 2008; 88: 324–32. 18689367 10.1093/ajcn/88.2.324

[28] Su CT , Ng HS , Yang AL , Lin CY. Psychometric evaluation of the Short Form 36 Health Survey (SF-36) and the World Health Organization Quality of Life Scale Brief Version (WHOQOL-BREF) for patients with schizophrenia. Psychol Assess 2014; 26: 980–9. 24796341 10.1037/a0036764

[29] John E.Ware J , Sherbourne CD. The MOS 36-item short-form health survey (SF-36): conceptual framework and item selection. Med Care 1992; 30: 473–83. 1593914

[30] Coates J , Swindale A , Bilinsky P. Household Food Insecurity Access Scale (HFIAS) for Measurement of Food Access: Indicator Guide: Version 3 . 2007. Food and Nutrition Technical Assistance Project (FANTA), 2007.

[31] Castell GS , Rodrigo CP , de la Cruz JN , Bartrina JA. Household food insecurity access scale (HFIAS). Nutr Hosp 2015; 31: 272–8. 25719795 10.3305/nh.2015.31.sup3.8775

[32] Flego A , Herbert J , Waters E , Gibbs L , Swinburn B , Reynolds J , et al. Jamie’s Ministry of Food: quasi-experimental evaluation of immediate and sustained impacts of a cooking skills program in Australia. PLoS One 2014; 9: e114673. 25514531 10.1371/journal.pone.0114673 PMC4267737

[33] Schulz KF , Altman DG , Moher D. CONSORT 2010 statement: updated guidelines for reporting parallel group randomised trials. J Pharmacol Pharmacother 2010; 1: 100–7. 21350618 10.4103/0976-500X.72352 PMC3043330

[34] Braun V , Clarke V. Using thematic analysis in psychology. Qual Res Psychol 2006; 3: 77–101.

[35] Association W M. World Medical Association Declaration of Helsinki: ethical principles for medical research involving human subjects. JAMA 2013; 310: 2191–4. 24141714 10.1001/jama.2013.281053

[36] Bradley T , Campbell E , Dray J , Bartlem K , Wye P , Hanly G , et al. Systematic review of lifestyle interventions to improve weight, physical activity and diet among people with a mental health condition. Syst Rev 2022; 11: 198. 36085250 10.1186/s13643-022-02067-3 PMC9462072

[37] Barch DM. The relationships among cognition, motivation, and emotion in schizophrenia: how much and how little we know. Schizophr Bull 2005; 31: 875–81. 16079388 10.1093/schbul/sbi040

[38] Killaspy H , Banerjee S , King M , Lloyd M. Prospective controlled study of psychiatric out-patient non-attendance: characteristics and outcome. Br J Psychiatry 2000; 176: 160–5. 10755054 10.1192/bjp.176.2.160

[39] Rössler W. The stigma of mental disorders: a millennia-long history of social exclusion and prejudices. Embo Rep 2016; 17: 1250–3. 27470237 10.15252/embr.201643041 PMC5007563

[40] Regev S , Josman N , Mendelsohn A. Looking beyond the laboratory: exploring behavioral and eye-fixation patterns in individuals with severe mental illness during a real-life supermarket task. Sci Progress 2023; 106: 00368504231160415. 10.1177/00368504231160415 PMC10450313 36919454

