# Supplementary material for: Schizophrenia, Nutrition and Choices in Kilojoules (SNaCK): protocol for a feasibility and acceptability randomised controlled trial of two dietary interventions
Source: BJPsych Open. 2025 Jul 22;11(4):e159. doi: 10.1192/bjo.2025.10070 (PMC12303830; doi:10.1192/bjo.2025.10070)
Supplement: Johnston et al. supplementary material 1 — Johnston et al. supplementary material [file S2056472425100707sup001.docx]

Supplementary File 1: Adherence checklists

**SNaCK Study Control Checklist**  **Participant ID/ Name:**

|  | Week 1 | Week 2 | Week 3 | Week 4 |
| --- | --- | --- | --- | --- |
| How much of the $100 supermarket voucher did you use to purchase food? | ❑ None  ❑ Some  ❑ All | ❑ None  ❑ Some  ❑ All | ❑ None  ❑ Some  ❑ All | ❑ None  ❑ Some  ❑ All |
| If you purchased food items, what did you purchase*?*  *(Select all that apply)* | ❑ Ingredients or food items to prepare meals at home  ❑ Fresh/frozen preprepared meals for reheating in the microwave  ❑Other_____________ | ❑ Ingredients or food items to prepare meals at home  ❑ Fresh/frozen preprepared meals for reheating the microwave  ❑ Other______ | ❑ Ingredients or food items to prepare meals at home  ❑ Fresh/frozen preprepared meals for reheating the microwave  ❑ Other___________ | ❑ Ingredients or food items to prepare meals at home  ❑ Fresh/frozen preprepared meals for reheating the microwave  ❑ Other__________ |
| Did you eat all/finish your meals this week? | ❑ *Yes* ❑ *No*  *If not, what happened to the leftovers/waste? (Select all that apply)*  ❑ *Someone else ate it*  ❑ *It went in the bin*  ❑ *I saved it for later*  ❑ Other____________ | ❑ *Yes* ❑ *No*  *If not, what happened to the leftovers/waste? (Select all that apply)*  ❑ *Someone else ate it*  ❑ *It went in the bin*  ❑ *I saved it for later*  ❑ Other____________ | ❑ *Yes* ❑ *No*  *If not, what happened to the leftovers/waste? (Select all that apply)*  ❑ *Someone else ate it*  ❑ *It went in the bin*  ❑ *I saved it for later*  ❑ Other___________ | ❑ *Yes* ❑ *No*  *If not, what happened to the leftovers/waste? (Select all that apply)*  ❑ *Someone else ate it*  ❑ *It went in the bin*  ❑ *I saved it for later*  ❑ Other____________ |
| How difficult was meal preparation this week? | ❑ Easy  ❑ Moderate  ❑ Hard | ❑ Easy  ❑ Moderate  ❑ Hard | ❑ Easy  ❑ Moderate  ❑ Hard | ❑ Easy  ❑ Moderate  ❑ Hard |
| Did anyone help you prepare your food? | ❑ Yes  ❑ No | ❑ Yes  ❑ No | ❑ Yes  ❑ No | ❑ Yes  ❑ No |

**SNaCK Study Meal Kit Checklist**  Participant ID/ Name: Week: One/Two/Three/Four

| Name of Meal  *(on recipe card)* | **Recipe 1** | | **Recipe 2** | | **Recipe 3** | | **Recipe 4** | |
| --- | --- | --- | --- | --- | --- | --- | --- | --- |
| Did you follow the recipe exactly? | ❑ Yes ❑ No  *If not, what did you do different?* | | ❑ Yes ❑ No  *If not, what did you do different?* | | ❑ Yes ❑ No  *If not, what did you do different?* | | ❑ Yes ❑ No  *If not, what did you do different?* | |
| How difficult was the meal preparation? | ❑ Easy ❑ Moderate ❑ Hard | | ❑ Easy ❑ Moderate ❑ Hard | | ❑ Easy ❑ Moderate ❑ Hard | | ❑ Easy ❑ Moderate ❑ Hard | |
| Did anyone help you make the recipe? | ❑ Yes ❑ No | | ❑ Yes ❑ No | | ❑ Yes ❑ No | | ❑ Yes ❑ No | |
| Did you split the recipe into 2 serves? | ❑ Yes ❑ No | | ❑ Yes ❑ No | | ❑ Yes ❑ No | | ❑ Yes ❑ No | |
| Days meal kit meals eaten | 1^st^ serve  ***Day:***  ***Date:*** | 2^nd^ serve  ***Day:***  ***Date:*** | 1^st^ serve  ***Day:***  ***Date:*** | 2^nd^ serve  ***Day:***  ***Date:*** | 1^st^ serve  ***Day:***  ***Date:*** | 2^nd^ serve  ***Day:***  ***Date:*** | 1^st^ serve  ***Day:***  ***Date:*** | 2^nd^ serve  ***Day:***  ***Date:*** |
| Did you eat any of the provided meal kit today?  (Tick amount eaten of the meal served.) | ❑ None  ❑ Some  ❑ All  *If some, how much*? | ❑ None  ❑ Some  ❑ All  *If some, how much*? | ❑ None  ❑ Some  ❑ All  *If some, how much*? | ❑ None  ❑ Some  ❑ All  *If some, how much*? | ❑ None  ❑ Some  ❑ All  *If some, how much*? | ❑ None  ❑ Some  ❑ All  *If some, how much*? | ❑ None  ❑ Some  ❑ All  *If some, how much*? | ❑ None  ❑ Some  ❑ All  *If some, how much*? |

**SNaCK Study Preprepared Meals Adherence Checklist**  Participant ID/ Name: Week: One/Two/Three/Four

|  | **Meal 1**  *Day:*  *Date:* | | **Meal 2**  *Day:*  *Date:* | | **Meal 3**  *Day:*  *Date:* | | **Meal 4**  *Day:*  *Date:* | | **Meal 5**  *Day:*  *Date:* | | **Meal 6**  *Day:*  *Date:* | | | **Meal 7**  *Day:*  *Date:* | | |
| --- | --- | --- | --- | --- | --- | --- | --- | --- | --- | --- | --- | --- | --- | --- | --- | --- |
| Did you consume any of the preprepared meals today? | ❑ Yes Entrée  ❑ Yes Main meal  ❑ No, if not why_________ | | ❑ Yes Entrée  ❑ Yes Main meal  ❑ No, if not why_________ | | ❑ Yes Entrée  ❑ Yes Main meal  ❑ No, if not why_________ | | ❑ Yes Entrée  ❑ Yes Main meal  ❑ No, if not why_________ | | ❑ Yes Entrée  ❑ Yes Main meal  ❑ No, if not why_________ | | ❑ Yes Entrée  ❑ Yes Main meal  ❑ No, if not why_________ | | | ❑ Yes Entrée  ❑ Yes Main meal  ❑ No, if not why_________ | | |
| Name of Entrée and Meal- as per menu | *e.g. Hearty Beef Casserole (450g* | |  | |  | |  | |  | |  | | |  | | |
| How much of your meal did you eat? | Entrée  ❑None  ❑Some  ❑ All  *If some,*  *how much? ________* | Main  ❑None  ❑Some  ❑ All  *If some,*  *how much?*  *_______* | Entrée  ❑None  ❑ Some  ❑ All  *If some, how*  *much?*  *___________* | Main  ❑None  ❑Some  ❑ All  *If some,*  *how much? _________* | Entrée  ❑None  ❑ Some  ❑ All  *If some, how*  *much?*  *_________* | Main  ❑None  ❑Some  ❑ All  *If some,*  *how much? _____­­­____* | Entrée  ❑None  ❑ Some  ❑ All  *If some,*  *how much? ________* | Main  ❑None  ❑Some  ❑ All  *If some,*  *how much? ________* | Entrée  ❑None  ❑ Some  ❑ All  *If some,*  *how*  *much? ________* | Main  ❑None  ❑Some  ❑ All  *If some,*  *how much? ________* | Entrée  ❑None  ❑ Some  ❑ All  *If some,*  *how*  *much? _____­­­___* | Main  ❑None  ❑Some  ❑ All  *If some,*  *how*  *much? ________* | Entrée  ❑None  ❑ Some  ❑ All  *If some,*  *how*  *much? ________* | | Main  ❑None  ❑Some  ❑ All  *If some,*  *how*  *much? ________* |  |
| If you didn’t eat ***all*** of your entrée or meal, what happened to the leftovers? | ❑ Someone else ate it  ❑ I saved it for later  ❑ It went in the bin or remained uneaten  ❑ Other ____________ | | ❑ Someone else ate it  ❑ I saved it for later  ❑ It went in the bin or remained uneaten  ❑ Other ____________ | | ❑ Someone else ate it  ❑ I saved it for later  ❑ It went in the bin or remained uneaten  ❑ Other ____________ | | ❑ Someone else ate it  ❑ I saved it for later  ❑ It went in the bin or remained uneaten  ❑ Other ____________ | | ❑ Someone else ate it  ❑ I saved it for later  ❑ It went in the bin or remained uneaten  ❑ Other ____________ | | ❑ Someone else ate it  ❑ I saved it for later  ❑ It went in the bin or remained uneaten  ❑ Other ____________ | | | ❑ Someone else ate it  ❑ I saved it for later  ❑ It went in the bin or remained uneaten  ❑ Other ____________ | | |
| How difficult was the meal preparation? | ❑ Easy  ❑ Moderate  ❑ Hard | | ❑ Easy  ❑ Moderate  ❑ Hard | | ❑ Easy  ❑ Moderate  ❑ Hard | | ❑ Easy  ❑ Moderate  ❑ Hard | | ❑ Easy  ❑ Moderate  ❑ Hard | | ❑ Easy  ❑ Moderate  ❑ Hard | | | ❑ Easy  ❑ Moderate  ❑ Hard | | |
| Did anyone help you prepare your meal? | ❑ Yes  ❑ No | | ❑ Yes  ❑ No | | ❑ Yes  ❑ No | | ❑ Yes  ❑ No | | ❑ Yes  ❑ No | | ❑ Yes  ❑ No | | | ❑ Yes  ❑ No | | |
