# Supplementary material for: Schizophrenia, Nutrition and Choices in Kilojoules (SNaCK): protocol for a feasibility and acceptability randomised controlled trial of two dietary interventions
Source: BJPsych Open. 2025 Jul 22;11(4):e159. doi: 10.1192/bjo.2025.10070 (PMC12303830; doi:10.1192/bjo.2025.10070)
Supplement: Johnston et al. supplementary material 2 — Johnston et al. supplementary material [file S2056472425100707sup002.docx]

Supplementary File 2: Acceptability Surveys

**Acceptability Survey – Meal Kits**

| **Participant question** | **1** | **2** | **3** | **4** | **5** |
| --- | --- | --- | --- | --- | --- |
| Did you like or dislike the meal kits? | Strongly dislike | Dislike | No opinion | Like | Strongly like |
| *What were the main reasons you liked/disliked the meal kits?* |  | | | | |
| How much effort did it take to prepare the meal kits? | Huge effort | A lot of effort | No opinion | A little effort | No effort at all |
| *What parts of the meal kits required the most effort? And why?* |  | | | | |
| I felt that receiving the meal kits was ethical and aligned with my values | Strongly disagree | Disagree | No opinion | Agree | Strongly Agree |
| *If you disagree, in what way did it not align with your values?* |  | | | | |
| It was clear to me what would be involved with using the meal kits | Strongly disagree | Disagree | No opinion | Agree | Strongly Agree |
| *If you disagree, what part of using the meal kits was not clear to you?* |  | | | | |
| Using the meal kits interfered with my routines | Strongly disagree | Disagree | No opinion | Agree | Strongly Agree |
| *If agree, in what way did it interfere?* |  | | | | |
| I felt that using the meal kits would likely improve my health | Strongly disagree | Disagree | No opinion | Agree | Strongly Agree |
| *Why/Why not?* |  | | | | |
| I felt confident that I could complete all that was required for me to use the meal kits | Strongly disagree | Disagree | No opinion | Agree | Strongly Agree |
| *If you disagree, what parts of the meal kits were you least confident about?* |  | | | | |
| Did you feel safe whilst using the meal kits? | Often felt unsafe | Sometimes felt unsafe | No opinion | Mostly felt safe | Always felt safe |
| *If no, please describe what parts of using the meal kits made you feel this way* |  | | | | |
| How acceptable was it for you to use the meal kits? | Completely unacceptable | Unacceptable | No opinion | Acceptable | Completely Acceptable |

**Acceptability Survey – Pre-prepared Meals**

| Did you like or dislike the pre-prepared meals? | Strongly dislike | Dislike | No opinion | Like | Strongly like |
| --- | --- | --- | --- | --- | --- |
| *What were the main reasons you liked/disliked the pre-prepared meals?* |  | | | | |
| How much effort did it take to prepare the pre-prepared meals? | Huge effort | A lot of effort | No opinion | A little effort | No effort at all |
| *What parts of the pre-prepared meals required the most effort? And why?* |  | | | | |
| I felt that receiving the pre-prepared meals was ethical and aligned with my values | Strongly disagree | Disagree | No opinion | Agree | Strongly Agree |
| *If you disagree, in what way did it not align with your values?* |  | | | | |
| It was clear to me what would be involved with using the pre-prepared meals | Strongly disagree | Disagree | No opinion | Agree | Strongly Agree |
| *If you disagree, what part of using the pre-prepared meals was not clear to you?* |  | | | | |
| Using the pre-prepared meals interfered with my routines | Strongly disagree | Disagree | No opinion | Agree | Strongly Agree |
| *If agree, in what way did it interfere?* |  | | | | |
| I felt that using the pre-prepared meals would likely improve my health | Strongly disagree | Disagree | No opinion | Agree | Strongly Agree |
| *Why/Why not?* |  | | | | |
| I felt confident that I could complete all that was required for me to use the pre-prepared meals | Strongly disagree | Disagree | No opinion | Agree | Strongly Agree |
| *If you disagree, what parts of the pre-prepared meals were you least confident about?* |  | | | | |
| Did you feel safe whilst using the pre-prepared meals? | Often felt unsafe | Sometimes felt unsafe | No opinion | Mostly felt safe | Always felt safe |
| *If no, please describe what parts of using the pre-prepared meals made you feel this way* |  | | | | |
| How acceptable was it for you to use the pre-prepared meals? | Completely unacceptable | Unacceptable | No opinion | Acceptable | Completely Acceptable |
