# Supplementary material for: Schizophrenia, Nutrition and Choices in Kilojoules (SNaCK): protocol for a feasibility and acceptability randomised controlled trial of two dietary interventions
Source: BJPsych Open. 2025 Jul 22;11(4):e159. doi: 10.1192/bjo.2025.10070 (PMC12303830; doi:10.1192/bjo.2025.10070)
Supplement: Johnston et al. supplementary material 3 — Johnston et al. supplementary material [file S2056472425100707sup003.docx]

Supplementary File 3: Risk Identification, Evaluation and Management Plan

| **Consequence** | **Response To Risk** |
| --- | --- |
| \| **Likelihood** \| **Negligible** \| **Minor** \| **Moderate** \| **Major** \| **Extreme** \| \| --- \| --- \| --- \| --- \| --- \| --- \| \| Almost Certain \|  \|  \|  \|  \|  \| \| Likely \|  \|  \|  \|  \|  \| \| Possible \|  \|  \|  \|  \|  \| \| Unlikely \|  \|  \|  \|  \|  \| \| Rare \|  \|  \|  \|  \|  \| | \|  \| Very High \| Immediate action required \| \| --- \| --- \| --- \| \|  \| High \| Urgent attention or investigation required \| \|  \| Medium \| Require specific attention \| \|  \| Low \| Manage through routine procedures \| |

**Risk Identification, Evaluation and Management Plan**

|  | Risk | Description | Possible Effects | | | Risk Management strategies |
| --- | --- | --- | --- | --- | --- | --- |
|  |  |  | Likelihood | Consequence | Rating |  |
| 1. | Psychological discomfort during interview | Participants may experience psychological discomfort when answering questions in the clinical interview | Possible | Minor-moderate | Medium | The PICF clearly states the potential risk of discomfort.  Recruitment of experienced mental health clinicians who will be able to minimise and manage discomfort.  Participants will be clinically assessed at baseline, and end point. We will also see participants once a week face-to-face to collect checklists and conduct physical measures as well as check they have received their meal intervention as per their order and preference.  Any discomfort reported or observed during these visits will be addressed by the research team.  Clinicians will direct and assist participants to gain support if required. |
| 2. | Inconvenience of participating in the trial | Participants may be inconvenienced by time taken to participate in the trial. | Possible | Negligible | Low | The PICF clearly states the battery of clinical assessments to be completed and the approximate time and frequency for clinical assessment visits.  Participants will be given as many breaks as necessary throughout the clinical assessment visits.  Participants will be reminded that the trial is voluntary and they can withdraw at any time. |
| 3. | History of self-harm/suicidal ideation | Participant expresses suicidal ideation. | Possible | Moderate-severe | High | Recruitment of experienced mental health clinicians who are trained in conducting risk assessment and managing high risk situations.  Research staff will have access to a clinically trained senior staff including a Project Manager and Chief Investigator who will assist research staff to conduct risk assessment and implement risk management plan if required i.e. notifying treating team and following advice from the treating team in managing the situation.  Previously identified high risk patients and recent risk assessments will be discussed at weekly team meetings and their management reviewed by senior research staff (including Project Manager and Chief Investigator).  Research staff will be given support and feedback on risk assessments and their management to improve skills throughout the project. |
| 4. | Home visits | Participants may be seen at home rather than in the clinic.  Individuals living with schizophrenia can often experience hallucinations and delusions which could result in unpredictable behaviour. | Possible | Minor-Moderate | Medium-High | Referrals will be received through Community care Units who have 24 hour support. Participants will be seen in either their Unit or clinic room on site if preferred, the choice is theirs.  Research staff will liaise with the treating team to assess any safety risks within the home environment prior to first face to face contact with the participant.  Staff will be required to carry a mobile phone for all visits.  Research staff will adhere to a sign in/out policy and advise the Research Manager of the address they will be attending.  Any incidents from a home visit will be reported to the Research Manager and Chief Investigator and documented in the CRF or if required reported to Metro South HREC. |
| 5. | Food preparation | Participants living with schizophrenia may experience cognitive decline which may affect their ability to prepare meals in a safe manner | Possible | Minor – moderate | Low – medium | Research staff will discuss safety education related to food preparation at each face-to-face visit paying particular attention to safety strategies to prevent burns and cuts. |
| 6. | Storing of food items before cooking and after cooking | Participants living with schizophrenia may experience cognitive decline which may affect their ability to store foods correctly | Possible | Minor – moderate | Medium | Research staff will provide food safety handouts at baseline assessments and reiterate this information at subsequent face to face visits, to encourage participants to store food correctly. |
| 7. | Healthy food choices | Participants living with schizophrenia may experience cognitive decline which may affect their ability to choose healthy food options | Possible | Moderate | Medium | Research staff will provide healthy food handouts at baseline assessments and reiterate this information at subsequent face to face visits, to encourage participants to maintain a healthy diet. |
